# Predictive models for kidney disease: improving global outcomes (KDIGO) defined acute kidney injury in UK cardiac surgery

**DOI:** 10.1186/s13054-014-0606-x

**Published:** 2014-11-20

**Authors:** Kate Birnie, Veerle Verheyden, Domenico Pagano, Moninder Bhabra, Kate Tilling, Jonathan A Sterne, Gavin J Murphy, on behalf of the UK AKI in Cardiac Surgery Collaborators

**Affiliations:** School of Social and Community Medicine, University of Bristol, Bristol, UK; Department of Cardiovascular Sciences, University of Leicester, Leicester NIHR Cardiovascular Biomedical Research Unit, Clinical Sciences Wing, Glenfield General Hospital, Leicester, LE3 9QP UK; Quality and Outcomes Research Unit, University Hospitals, Birmingham & School of Experimental Medicine, University of Birmingham, Birmingham, UK; University Hospitals of Birmingham NHS Foundation Trust, Birmingham, UK

## Abstract

**Introduction:**

Acute kidney injury (AKI) risk prediction scores are an objective and transparent means to enable cohort enrichment in clinical trials or to risk stratify patients preoperatively. Existing scores are limited in that they have been designed to predict only severe, or non-consensus AKI definitions and not less severe stages of AKI, which also have prognostic significance. The aim of this study was to develop and validate novel risk scores that could identify all patients at risk of AKI.

**Methods:**

Prospective routinely collected clinical data (*n* = 30,854) were obtained from 3 UK cardiac surgical centres (Bristol, Birmingham and Wolverhampton). AKI was defined as per the Kidney Disease: Improving Global Outcomes (KDIGO) Guidelines. The model was developed using the Bristol and Birmingham datasets, and externally validated using the Wolverhampton data. Model discrimination was estimated using the area under the ROC curve (AUC). Model calibration was assessed using the Hosmer–Lemeshow test and calibration plots. Diagnostic utility was also compared to existing scores.

**Results:**

The risk prediction score for any stage AKI (AUC = 0.74 (95% confidence intervals (CI) 0.72, 0.76)) demonstrated better discrimination compared to the Euroscore and the Cleveland Clinic Score, and equivalent discrimination to the Mehta and Ng scores. The any stage AKI score demonstrated better calibration than the four comparison scores. A stage 3 AKI risk prediction score also demonstrated good discrimination (AUC = 0.78 (95% CI 0.75, 0.80)) as did the four comparison risk scores, but stage 3 AKI scores were less well calibrated.

**Conclusions:**

This is the first risk score that accurately identifies patients at risk of any stage AKI. This score will be useful in the perioperative management of high risk patients as well as in clinical trial design.

## Introduction

Acute kidney injury (AKI) is a common and severe complication of cardiac surgery affecting up to 30% of all patients and increasing mortality up to fourfold [1,2]. No effective treatment has been identified despite over 70 randomised controlled trials (RCTs) of proposed renoprotective interventions [3,4]. Systematic reviews have documented important limitations in these trials such as the enrolment of low or mixed AKI-risk patient cohorts, small sample sizes, and low statistical power [3,4]. These are important sources of bias that will have increased the likelihood of negative trial results. Recent recommendations on the design of trials in AKI suggest that these limitations may be countered by cohort enrichment [5], whereby the enrolment of patients with higher event rates will permit targeting of interventions to those patients populations most likely to benefit, smaller sample sizes, and increased study power. AKI risk prediction scores are an objective, transparent means of cohort enrichment but are not widely used. This is because existing scores have been developed principally to identify patients at risk of renal replacement therapy (RRT) which is rare and not less severe AKI, which is also associated with poor prognosis but occurs with greater frequency [6]. There is also uncertainty as to the generalizability of these scores. Only two published scores, The Cleveland clinic score [7], and the Mehta score [8] have been independently validated, and neither has demonstrated adequate discrimination and calibration in non-North American patient populations [9,10].

The aim of this study was to develop and externally validate two novel risk scores that could be used for cohort enrichment in clinical trials of renoprotective interventions in cardiac surgery. We used the Kidney Disease: Improving Global Outcomes (KDIGO) guidelines AKI definition [11] as this reconciles important differences between the two earlier consensus definitions; Acute Kidney Injury Network (AKIN) [12] and risk, injury, failure, loss, end stage (RIFLE) [13]. The prognostic utility of this score has only been demonstrated thus far in a single centre [14]. To support the validity and utility of the risk scores our first objective was therefore to demonstrate the utility of the KDIGO definition in a large multicentre cohort. Next, we developed two new scores based on preoperative variables; one to identify all patients at risk of AKI (KDIGO stages 1 to 3), and the second to identify only those patients at risk of severe AKI (KDIGO stage 3). Finally we compared the diagnostic utility of these new scores with four published risk scores.

## Methods

This study was approved by the South West research ethics committee under reference 11/SW/0075. The requirement for written informed consent was waived. Our research objectives and methods were specified prior to execution.

### Study population

This retrospective cohort study used routinely collected data from the patient analysis and tracking system (PATS), from three UK hospitals. We obtained prospectively collected data on all adult cardiac patients for the following periods: 1996 to 2010 at University Hospitals of Bristol, 2002 to 2010 at University Hospitals of Birmingham National Health Service (NHS) Foundation Trust, and 2004 to 2010 at Wolverhampton Heart and Lung Centre. Our analyses included all patients aged ≥16 years, who underwent cardiac surgery, with or without cardiopulmonary bypass (CBP), and including those who underwent surgery to the thoracic aorta; we excluded patients already on renal replacement therapy (RRT) or who had received a kidney transplants, and patients who died in theatre.

Definitions of perioperative variables used were consistent across the three sites, and are as specified by the UK National Adult Cardiac Surgery Audit (NACSA) [15]. NACSA is a component of the UK cardiac surgery national quality assurance programme, whereby a defined set of perioperative data are collected prospectively by the anaesthetist, surgeon, and ICU, high-dependency unit (HDU), and ward nurses on the PATS databases. These data are submitted to the National Institute Clinical Outcome Research (NICOR) for analysis and the generation of risk-adjusted outcome data [16]. The data undergo routine internal quality assurance prior to submission as well as external data quality assessment by NICOR. The NACSA Annual Report for 2010 indicated that these three centres are among the best units nationally for data quality [17]. PATS data were linked using name, hospital number, and date of birth, to institutional biochemistry databases that record serial serum creatinine results, to identify those patients who developed AKI. During the study serum creatinine was measured using the Jaffe method at all three centres. Wolverhampton and Birmingham used the Roche Modular system (Roche Diagnostics, Ltd, Lewes, UK). Bristol used the Olympus Diagnostics System AU640 or AU2700 (Olympus Diagnostic Systems, Southall, UK). The assays were calibrated using the manufacturers’ controls with inter-institutional diagnostic accuracy monitored by a national (UK) quality assessment scheme.

### Definitions

#### Measurement of acute kidney injury

AKI was classified according to the KDIGO guidelines [11]. Stage-1 AKI was defined as an increase from baseline of ≥26 μmol/L of postoperative creatinine or an increase of 1.5 to 1.9 times the preoperative creatinine within 7 days; stage 2 was an increase of 2.0 to 2.9 times the preoperative creatinine; stage-3 AKI was an increase ≥3 times the preoperative creatinine or an increase to ≥354 μmol/L or when the patient commenced RRT. The RRT was administered for uraemia, volume overload, or biochemical abnormalities, according to institutional protocols. Urine output data were not available and therefore not used in our AKI definition. The baseline creatinine value was defined as the preoperative value obtained closest to the date of the operation. Cases where the baseline data were missing, most commonly emergency and salvage patients, were not included in the complete case analyses.

#### Demographic factors

Information was extracted from PATS on age (grouped into three categories: <60; 60 to 74; or ≥75 years), sex, body mass index (BMI) (grouped into five categories: <20.0; 20.0 to 24.9; 25.0 to 29.9; 30.0 to 34.9; or ≥35.0 kg/m^2^) and smoking status (never smoked; ex-smoker; or current smoker).

#### Preoperative factors

Information on prespecified factors was obtained from PATS for the three centres. The presence of angina was grouped according to the Canadian Cardiovascular Society (CCS) categories: no angina, CCS 1 (ordinary activity does not cause angina); CCS 2 (slight limitation); CCS 3 (moderate limitation); or CCS 4 (inability to carry out any physical activity without discomfort). Dyspnoea was grouped according to the New York Heart Association (NYHA) functional classification: NYHA 1 (no symptoms and no limitation in ordinary physical activity); NHYA 2 (mild symptoms and slight limitation during ordinary activity); NYHA 3 (marked limitation in activity due to symptoms, comfortable only at rest); or NYHA 4 (severe limitations, experiences symptoms even while at rest). Previous myocardial infarction (MI) was categorised as; none; 1; or ≥2. Information was obtained on previous cardiac, vascular or thoracic surgery (0 and ≥1), the presence of diabetes, peripheral vascular disease, pulmonary disease, neurological disease, hypertension (treated or blood pressure (BP) >140/90) and preoperative haemoglobin (<10.0; 10.0 to 11.9; or ≥12.0 g/dL). Glomerular filtration rate (GFR) was estimated from the Cockcroft-Gault equation using the preoperative creatinine value obtained closest to the day of surgery, and grouped as: <30.0; 30.0 to 59.9; 60.0 to 89.9; or ≥90 μmol/L. Heparin or nitrates usage was grouped according to: none; within a week; or at operation. Any critical preoperative event was considered to be cardiogenic shock; preoperative intravenouse (IV) inotropes; or preoperative ventilation or intra-aortic balloon pump (IABP). The time between catheterisation and surgery was grouped as: within 24 hours; >24 hours for this admission; or >24 hours for a previous admission. Information was obtained on triple vessel disease, left main stem disease, ejection fraction (grouped as: good ≥50%; fair 30 to 49%; or poor <30%), operative priority (elective, urgent and emergency/salvage). Finally the cardiac procedures being undertaken were classified as coronary artery bypass graft surgery (CAGB) only; valve only; or CABG and valve and other/multiple. Procedures classed as other included major aortic surgery; left ventricular aneurysmectomy; atrial myxoma surgery; pulmonary embolectomy; epicardial pacemaker placement; pericardectomy; atrial septal defect closure; procedure for congenital conditions; acquired ventricular septal defect closure; pulmonary endarterectomy; atrial fibrillation ablation; myomectomy; cardiac surgery plus carotid endarterectomy or peripheral vascular procedures; or any other cardiothoracic procedure not listed above.

#### Outcomes following surgery

Information on pulmonary complications (reintubation and ventilation; full tracheostomy; need for CPAP or BIPAP; or prolonged ventilation >48 hours), infectious complications (sternal wound infection; leg wound infection; chest infection; or septicaemia or other infection), length of stay in hospital (in days) and death in hospital was obtained from PATS.

### Statistical analyses

The association between AKI and outcomes following surgery were modelled using logistic regression models. The data from Bristol and Birmingham were used to develop the prognostic models (development sample) and the models were validated on the data from Wolverhampton (validation sample). Any-stage AKI and stage-3 AKI were modelled using separate logistic regression models. Univariable associations were examined for all demographic and preoperative factors. Mutivariable associations were examined by entering all demographic and preoperative factors into a single model, which controlled for all these main effects, in a full model. Factors selected for the initial prognostic model were those from the multivariable full model with *P* <0.001. Factors selected for the more inclusive prognostic model were those from the full model with *P* <0.05. The area under the receiver operating characteristic (ROC) curve (AUC) was calculated for each model in the development and validation samples to quantify diagnostic utility. Model calibration was assessed using the Hosmer-Lemeshow test and calibration plots. Calibration plots of observed versus predicted values for AKI were analysed using linear regression to provide the slope and intercept where the closer the intercept to 0, and the closer the slope to 1, the better the calibration. The discrimination and calibration of the models were compared to two published AKI risk scores from US data [7,8], a recently published risk score from Australia [10] as well as a mortality risk score - the logistic Euroscore [18]. We were able to match all the variables for the Cleveland clinic score [7] except congestive heart failure and left ventricular ejection fraction <35% (data cut at <30%). For the Ng score [10] we were able to match all the variables with the exception of infective endocarditis. For the Mehta score [8] we were unable to match ethnicity in the development sample, and were unable to precisely match the field MI within 3 weeks, or the type of valve procedure (mitral versus aortic).

We conducted sensitivity analyses, first by considering the inclusion of two-way interactions in our score, and second, by using multiple imputations by chained equations [19,20] to account for missing data. The analysis assumes any systematic difference between the missing values and the observed values can be explained by differences in observed data. We used the ice command [21] in Stata to impute confounder and outcome missing data. Variables that may help explain the missing data (for example, demographic information, preoperative factors, AKI status and outcomes following surgery) were included in the imputation model. The missing values were sampled from their predictive distribution, based on the observed data. Standard regression analyses were used to fit the model of interest to each of the imputed datasets. Ten cycles of regression were carried out and 20 datasets imputed. All 20 estimated associations were combined to give one overall estimated association of interest. Standard error was calculated using the Rubin rules [22,23]. These rules took into account the variability in results between the imputed datasets, indicating the uncertainty of the missing values. All analyses were carried out in StataTM version 13.

### Online calculator

We constructed a web-based calculator for the any-stage AKI risk score [24].

## Results

### Characteristics of patients

Data were available from 18,686 patients from Bristol and 7,306 patients from Birmingham (the developmental sample) and 4,862 patients from Wolverhampton (the validation sample). Missing data for each centre are reported in Table 1. The most common field that was missing was the baseline Hb. This was missing in 39% of the Birmingham data, and predominantly in the years prior to 2004. Emergency/salvage surgery were more likely to have missing preoperative creatinine (Bristol 10%, Birmingham 14%, Wolverhampton 3% missing) compared to elective or urgent surgery (Bristol 1%, Birmingham 1%, Wolverhampton 0% missing). The complete case analysis included 20,995 patients, of whom 12,435 were from Bristol, 4,092 were from Birmingham and 4,468 were from Wolverhampton (Table 2).

**Table 1:** **Number and percentage missing for each variable by centre**

	**Bristol (n = 18,686)**		**Birmingham (n = 7,306)**		**Wovlerhampton (n = 4,862)**	
**Variable**	**Number**	**%**	**Number**	**%**	**Number**	**%**
Age	0	0.0	0	0.0	0	0.0
Sex	2	0.0	0	0.0	0	0.0
Body mass index	341	1.8	29	0.4	1	0.0
Smoking status	114	0.6	28	0.4	1	0.0
Angina	79	0.4	15	0.2	0	0.0
Dyspnoea	93	0.5	20	0.3	0	0.0
Previous MI	54	0.3	37	0.5	10	0.2
Previous operations	15	0.1	9	0.1	0	0.0
Preoperative diabetes	75	0.4	15	0.2	0	0.0
Peripheral vascular disease	67	0.4	16	0.2	0	0.0
Pulmonary disease	65	0.4	18	0.3	0	0.0
Neurological disease	59	0.3	14	0.2	0	0.0
Hypertension	130	0.7	29	0.4	1	0.0
Haemoglobin	1,298	7.0	2,846	39.0	28	0.6
Baseline serum creatinine	282	1.5	66	0.9	19	0.4
GFR	537	2.9	86	1.2	19	0.4
Heparin or nitrates	51	0.3	18	0.3	0	0.0
Critical preoperative event	0	0.0	12	0.2	0	0.0
Catheterisation to surgery	1,447	7.7	398	5.5	242	5.0
Triple vessel disease	938	5.0	252	3.5	85	1.8
Left main stem disease	2,286	12.2	245	3.4	83	1.7
Ejection fraction	493	2.6	86	1.2	0	0.0
Operative priority	23	0.1	0	0.0	0	0.0
Cardiac procedures	0	0.0	0	0.0	0	0.0
Postoperative creatinine	282	1.5	66	0.9	84	1.7
Acute kidney injury	629	3.4	215	2.9	84	1.7
Acute kidney injury stage	629	3.4	215	2.9	84	1.7
Postoperative stay	10	0.1	0	0.0	0	0.0
Infective complication	1,564	8.4	61	0.8	96	2.0
Pulmonary complication	1,651	8.8	53	0.7	100	2.1
Died in hospital	1	0.0	6	0.1	0	0.0
Euroscore	129	0.7	48	0.7	6	0.1
Cleveland clinic score*	731	3.9	139	1.9	19	0.4
Mehta score*	369	2.0	101	1.4	29	0.6
Ng score*	613	3.3	92	1.3	20	0.4

*Approximation to scores, matching where data are available. MI, myocardial infaction.

**Table 2:** **Characteristics of patients by centre in the complete case data**

	**Bristol (n = 12,435)**	**Birmingham (n = 4,092)**	**Wolverhampton (n = 4,468)**
	**Number (%)**	**Number (%)**	**Number (%)**
**Demographic factors**			
Age, years			
<60	2,995 (24.1)	1,061 (25.9)	1,026 (23.0)
60 to 74	6,873 (55.3)	2,201 (53.8)	2,471 (55.3)
≥75	2,567 (20.6)	830 (20.3)	971 (21.7)
Sex			
Male	9,474 (76.2)	2,990 (73.1)	3,420 (76.5)
Female	2,961 (23.8)	1,102 (26.9)	1,048 (23.5)
Body mass index			
<20.0	482 (3.9)	112 (2.7)	87 (1.9)
20.0 to 24.9	3,206 (25.8)	1,003 (24.5)	1,057 (23.7)
25.0 to 29.9	5,685 (45.7)	1,737 (42.4)	2,020 (45.2)
30.0 to 34.9	2,365 (19.0)	893 (21.8)	967 (21.6)
35.0+	697 (5.6)	347 (8.5)	337 (7.5)
Smoking status			
Never smoked	3,970 (31.9)	1,351 (33.0)	1,633 (36.5)
Ex-smoker	7,072 (56.9)	2,427 (59.3)	2,414 (54.0)
Current smoker	1,393 (11.2)	314 (7.7)	421 (9.4)
**Preoperative factors**			
Angina^a^			
No angina	2,346 (18.9)	737 (18.0)	779 (17.4)
CCS 1	1,460 (11.7)	399 (9.8)	244 (5.5)
CCS 2	3,611 (29.0)	1,354 (33.1)	1,342 (30.0)
CCS 3	2,885 (23.2)	949 (23.2)	1,142 (25.6)
CCS 4	2,133 (17.2)	653 (16.0)	961 (21.5)
Dyspnoea^b^			
NYHA 1	2,797 (22.5)	786 (19.2)	926 (20.7)
NHYA 2	5,129 (41.2)	2,094 (51.2)	1,748 (39.1)
NYHA 3	3,895 (31.3)	928 (22.7)	1,353 (30.3)
NYHA 4	614 (4.9)	284 (6.9)	441 (9.9)
Previous MI			
0	7,786 (62.6)	2,671 (65.3)	2,453 (54.9)
1	3,783 (30.4)	1,145 (28.0)	1,620 (36.3)
≥2	866 (7.0)	276 (6.7)	395 (8.8)
Previous operations			
0	1,1940 (96.0)	3,895 (95.2)	4,352 (97.4)
1+	495 (4.0)	197 (4.8)	116 (2.6)
Preoperative diabetes			
No	10,243 (82.4)	3,176 (77.6)	3,399 (76.1)
Yes	2,192 (17.6)	916 (22.4)	1,069 (23.9)
Peripheral vascular disease			
No	11,388 (91.6)	3,536 (86.4)	3,665 (82.0)
Yes	1,047 (8.4)	556 (13.6)	803 (18.0)
Pulmonary disease			
No	10,991 (88.4)	3,499 (85.5)	3,926 (87.9)
Yes	1,444 (11.6)	593 (14.5)	542 (12.1)
Neurological disease			
No	11,303 (90.9)	3,790 (92.6)	4,077 (91.2)
Yes	1,132 (9.1)	302 (7.4)	391 (8.8)
Hypertension^c^			
No	4,400 (35.4)	1,307 (31.9)	1,673 (37.4)
Yes	8,035 (64.6)	2,785 (68.1)	2,795 (62.6)
Haemoglobin			
<10.0	218 (1.8)	91 (2.2)	71 (1.6)
10.0 to11.9	1,487 (12.0)	593 (14.5)	515 (11.5)
12.0+	10,730 (86.3)	3,408 (83.3)	3,882 (86.9)
GFR			
<30.0	436 (3.5)	102 (2.5)	54 (1.2)
30.0 to 59.9	4,592 (36.9)	1,460 (35.7)	1,059 (23.7)
60.0 to 89.9	5,458 (43.9)	1,741 (42.5)	1,887 (42.2)
90.0+	1,949 (15.7)	789 (19.3)	1,468 (32.9)
Heparin or nitrates			
None	10,724 (86.2)	3,595 (87.9)	4,133 (92.5)
Within a week	914 (7.4)	53 (1.3)	114 (2.6)
At operation	797 (6.4)	444 (10.9)	221 (4.9)
Critical preoperative event			
No	12,268 (98.7)	3,944 (96.4)	4,392 (98.3)
Yes	167 (1.3)	148 (3.6)	76 (1.7)
Catheter to surgery			
Within 24 h	299 (2.4)	38 (0.9)	84 (1.9)
>24 h this admission	6,825 (54.9)	1,019 (24.9)	971 (21.7)
>24 h previous admission	5,311 (42.7)	3,035 (74.2)	3,413 (76.4)
Triple vessel disease			
No	6,041 (48.6)	1,759 (43.0)	1,819 (40.7)
Yes	6,394 (51.4)	2,333 (57.0)	2,649 (59.3)
Left main stem disease			
No	10,208 (82.1)	3,162 (77.3)	3,422 (76.6)
Yes	2,227 (17.9)	930 (22.7)	1,046 (23.4)
Ejection fraction			
Good (50+ %)	9,111 (73.3)	2,758 (67.4)	2,910 (65.1)
Fair (30 to 49%)	2,668 (21.5)	1,143 (27.9)	1,192 (26.7)
Poor (<30%)	656 (5.3)	191 (4.7)	366 (8.2)
Operative priority			
Elective	7,350 (59.1)	2,798 (68.4)	3,173 (71.0)
Urgent	4,896 (39.4)	1,256 (30.7)	1,255 (28.1)
Emergency/salvage	189 (1.5)	38 (0.9)	40 (0.9)
Cardiac procedures			
CABG only	8,587 (69.1)	2,241 (54.8)	2,974 (66.6)
Valve only	1,838 (14.8)	644 (15.7)	622 (13.9)
CABG+valve	1,169 (9.4)	554 (13.5)	568 (12.7)
Other/multiple	841 (6.8)	653 (16.0)	304 (6.8)
**Acute kidney injury, %**			
None	9,495 (76.4)	3,238 (79.1)	3,370 (75.4)
Stage 1	2,164 (17.4)	560 (13.7)	706 (15.8)
Stage 2	330 (2.7)	89 (2.2)	124 (2.8)
Stage 3	446 (3.6)	205 (5.0)	268 (6.0)

^a^Canadian Cardiovascular Society (CCS) classes: 1 ordinary activity does not cause angina; 2 slight limitation; 3 marked limitation; 4 inability to carry out any physical activity without discomfort. ^b^New York Heart Association (NYHA) functional classification: 1 no symptoms and no limitation in ordinary physical activity; 2 mild symptoms and slight limitation during ordinary activity; 3 marked limitation in activity due to symptoms, comfortable only at rest; 4 severe limitations, experiences symptoms even while at rest. ^c^Hypertension was defined as treated or blood pressure >140/90. CABG, coronary artery bypass graft; GFR, glomerular filtration rate; Haemoglobin, g/dL; GFR, mL/min.

In the complete case data median age by centre was: Bristol 68 years (IQR 60, 74), Birmingham 67 years (59, 73) and Wolverhampton 67 years (60, 74). The majority of operations were performed on men (76.2% in Bristol, 73.1% in Birmingham and 76.5% in Wolverhampton). The most common procedure was CABG only (69.1% in Bristol, 54.8% in Birmingham and 66.6% in Wolverhampton). Mean (SD) baseline creatinine values by centre were Bristol 108.9 μmol/L (28.5), Birmingham 107.1 μmol/L (34.4) and Wolverhampton 94.8 μmol/L (28.4) Any stage of AKI affected 23.7% patients in the Bristol dataset, 20.9% patients in Birmingham and 24.6% patients in Wolverhampton. Stage-3 AKI was slightly more prevalent in the validation dataset (6.0%) compared to the development sample (3.6% in Bristol and 5.0% in Birmingham). The criteria by which patients were defined as having AKI are described in Table 3.

**Table 3:** **Total patients who met different acute kidney injury (AKI) criteria in the complete case data**

	**Ratio of postoperative to preoperative creatinine**	**Postoperative creatinine <354**		**Postoperative creatinine ≥354**	
		**Difference from baseline**			
		**<26**	**≥26**	**<26**	**≥26**
No RRT	<1.5	16,094^a^	2,152^b^	9^a^	11^d^
	1.5 to 1.9	1^b^	1,277^b^		33^d^
	2.0 to 2.9		543^c^		94^d^
	≥3.0		81^d^		95^d^
Commenced on RRT	<1.5	77^d^	71^d^		4^d^
	1.5 to 1.9		137^d^		7^d^
	2.0 to 2.9		155^d^		34^d^
	≥3.0		43^d^		77^d^

Creatinine measure in μmol/L. ^a^No AKI, total = 16,103; ^b^stage-1 AKI, total = 3,430; ^c^stage-2 AKI, total = 543; ^d^stage-3 AKI, total = 919. RRT, renal replacement therapy.

### KDIGO-defined AKI severity and outcome

AKI was associated with increased hospital stay (Table 4). Patients without AKI had a median postoperative hospital stay of 6 days in Bristol (IQR 5, 8) and Wolverhampton (5, 7), and 9 days (6, 13) in Birmingham (Table 4). For patients with stage-1 AKI, median postoperative stay increased to 9 days (IQR 7, 13) in Bristol, 13 (9, 19) in Birmingham and 8 (6, 13) in Wolverhampton. Patients with the most severe AKI (stages 2 and 3) had the longest postoperative stay. In the complete case data (n = 20,995) stage-1 AKI was associated with higher odds of infective and pulmonary complications and an almost five-fold increase in the odds of death in hospital (Table 5). Odds ratios for stage-3 AKI compared to no AKI were 7.6 (95% CI 6.6, 8.8) for infective complications, 9.4 (8.2, 10.8) for pulmonary complications and 81.2 (62.3, 106.0) for death in hospital. Over 80% of all deaths in hospital were preceded by AKI.

**Table 4:** **Length of stay following surgery by acute kidney injury (AKI) status**

	**Bristol**			**Birmingham**			**Wolverhampton**		
	**Numbe**	**Median**	**IQR**	**Number**	**Median**	**IQR**	**Number**	**Median**	**IQR**
**All patients**									
AKI									
None	9495	6	(5, 8)	3238	9	(6, 13)	3370	6	(5, 7)
Stage 1	2164	9	(7, 13)	560	13	(9, 19)	706	8	(6, 13)
Stage 2	330	11	(8, 16)	89	19	(12, 32)	124	10	(8, 17)
Stage 3	446	14	(10, 26)	205	19	(10, 42)	268	16	(9, 32)
**Patients who did not die in hospital**									
AKI									
None	9464	6	(5, 8)	3205	9	(7, 13)	3357	6	(5, 7)
Stage 1	2119	9	(7, 13)	544	13	(9, 18)	693	8	(6, 13)
Stage 2	313	11	(8, 16)	88	19	(12, 32)	122	10	(8, 17)
Stage 3	338	15	(10, 27)	133	25	(14, 43)	190	17	(11, 32)

**Table 5:** **Outcomes following surgery by acute kidney injury (AKI) status, data from all centres**

**AKI status**	**Total number**	**Infective complication**		**Pulmonary complication**		**Died in hospital**	
		**Number (%)**	**Odds ratio (95% CI)**	**Number (%)**	**Odds ratio (95% CI)**	**Number (%)**	**Odds ratio (95% CI)**
None	16103	1430 (8.9)	1	1810 (11.2)	1	77 (0.5)	1
Stage 1	3430	623 (18.2)	2.3 (2.1, 2.5)	793 (23.1)	2.4 (2.2, 2.6)	74 (2.2)	4.6 (3.3, 6.3)
Stage 2	543	133 (24.5)	3.3 (2.7, 4.1)	192 (35.4)	4.3 (3.6, 5.2)	20 (3.7)	8.0 (4.8, 13.1)
Stage 3	919	392 (42.7)	7.6 (6.6, 8.8)	500 (54.4)	9.4 (8.2, 10.8)	258 (28.1)	81.2 (62.3, 106.0)
*P*-value			<0.001		<0.001		<0.001

*P*-values for trend derived from logistic regression models.

### Model building for the clinical prediction score

A total of 23 factors were examined for their associations with AKI. Odds ratios for unadjusted and fully adjusted models are presented in Table 6 (for models of any-stage AKI) and Table 7 (for models of stage-3 AKI). In fully adjusted models, 15 factors were strongly associated with any-stage AKI (*P* <0.001) and were included in the initial prognostic model. A further five factors were associated with any-stage AKI at the level of *P* <0.05, and were added to the more inclusive model. For stage-3 AKI, eight factors were included in the initial prognostic model (*P* <0.001), a further three factors were included in the more inclusive prognostic model (*P* <0.05).

**Table 6:** **Associations of preoperative factors with any-stage acute kidney injury (AKI) in the development sample (Bristol and Birmingham)**

	**Number**	**Number (%)**	**Unadjusted**			**Fully adjusted**		
	**Total**	**Any AKI**	**Odds ratio**	**95% CI**	***P*** **-value**	**Odds ratio**	**95% CI**	***P*** **-value**
**Demographic factors**								
Age, years								
<60	4,056	485 (12.0)	1.00			1.00		
60 to 74	9,074	2,039 (22.5)	2.13	(1.92, 2.37)		1.53	(1.35, 1.74)	
≥75	3,397	1,270 (37.4)	4.40	(3.91, 4.94)	<0.001	2.21	(1.91, 2.57)	<0.001
Sex								
Male	12,464	2,891 (23.2)	1.00			1.00		
Female	4,063	903 (22.2)	0.95	(0.87, 1.03)	0.202	0.53	(0.48, 0.59)	<0.001
Body mass index								
<20.0	594	175 (29.5)	1.60	(1.33, 1.92)		0.80	(0.64, 0.99)	
20.0 to 24.9	4,209	931 (22.1)	1.08	(0.99, 1.19)		0.79	(0.71, 0.88)	
25.0 to -29.9	7,422	1,540 (20.7)	1.00			1.00		
30.0 to 34.9	3,258	854 (26.2)	1.36	(1.23, 1.49)		1.75	(1.57, 1.95)	
35.0+	1,044	294 (28.2)	1.50	(1.29, 1.73)	<0.001	2.11	(1.78, 2.50)	<0.001
Smoking status								
Never smoked	5,321	1,122 (21.1)	1.00			1.00		
Ex-smoker	9,499	2,315 (24.4)	1.21	(1.11, 1.31)		1.09	(1.00, 1.20)	
Current smoker	1,707	357 (20.9)	0.99	(0.87, 1.13)	<0.001	1.41	(1.22, 1.64)	<0.001
**Preoperative factors**								
Angina								
No angina	3,083	810 (26.3)	1.00			1.00		
CCS 1	1,859	409 (22.0)	0.79	(0.69, 0.91)		0.84	(0.73, 0.98)	
CCS 2	4,965	994 (20.0)	0.70	(0.63, 0.78)		0.79	(0.69, 0.90)	
CCS 3	3,834	870 (22.7)	0.82	(0.74, 0.92)		0.80	(0.70, 0.92)	
CCS 4	2,786	711 (25.5)	0.96	(0.86, 1.08)	<0.001	0.75	(0.64, 0.87)	0.002
Dyspnoea								
NYHA 1	3,583	610 (17.0)	1.00			1.00		
NHYA 2	7,223	1,465 (20.3)	1.24	(1.12, 1.38)		1.07	(0.95, 1.19)	
NYHA 3	4,823	1,360 (28.2)	1.91	(1.72, 2.13)		1.29	(1.14, 1.46)	
NYHA 4	898	359 (40.0)	3.25	(2.77, 3.81)	<0.001	1.37	(1.13, 1.66)	<0.001
Previous MI								
0	10,457	2,272 (21.7)	1.00			1.00		
1	4,928	1,174 (23.8)	1.13	(1.04, 1.22)		0.99	(0.90, 1.10)	
≥2	1,142	348 (30.5)	1.58	(1.38, 1.81)	<0.001	1.04	(0.88, 1.21)	0.879
Previous operations								
0	15,835	3,578 (22.6)	1.00			1.00		
1+	692	216 (31.2)	1.55	(1.32, 1.83)	<0.001	1.39	(1.15, 1.67)	0.007
Preoperative diabetes								
No	13,419	2,824 (21.0)	1.00			1.00		
Yes	3,108	970 (31.2)	1.70	(1.56, 1.86)	<0.001	1.41	(1.28, 1.55)	<0.001
Peripheral vascular disease								
No	14,924	3,237 (21.7)	1.00			1.00		
Yes	1,603	557 (34.7)	1.92	(1.72, 2.15)	<0.001	1.31	(1.16, 1.48)	<0.001
Pulmonary disease								
No	14,490	3,217 (22.2)	1.00			1.00		
Yes	2,037	577 (28.3)	1.38	(1.25, 1.54)	<0.001	1.07	(0.96, 1.20)	0.240
Neurological disease								
No	15,093	3,346 (22.2)	1.00			1.00		
Yes	1,434	448 (31.2)	1.60	(1.42, 1.80)	<0.001	1.14	(1.00, 1.30)	0.044
Hypertension								
No	5,707	1,028 (18.0)	1.00			1.00		
Yes	10,820	2,766 (25.6)	1.56	(1.44, 1.69)	<0.001	1.28	(1.17, 1.40)	<0.001
Haemoglobin								
<10.0	309	133 (43.0)	2.98	(2.37, 3.74)		1.58	(1.23, 2.04)	
10.0 to11.9	2,080	800 (38.5)	2.46	(2.23, 2.72)		1.67	(1.49, 1.87)	
12.0+	14,138	2,861 (20.2)	1.00		<0.001	1.00		<0.001
Glomerular filtration rate								
<30.0	538	283 (52.6)	5.64	(4.71, 6.75)		4.64	(3.73, 5.77)	
30.0 to 59.9	6,052	1,917 (31.7)	2.36	(2.17, 2.56)		2.25	(2.03, 2.48)	
60.0 to 89.9	7,199	1,184 (16.4)	1.00			1.00		
90.0+	2,738	410 (15.0)	0.89	(0.79, 1.01)	<0.001	0.82	(0.71, 0.95)	<0.001
Heparin or nitrates								
None	14,319	3,086 (21.6)	1.00			1.00		
Within a week	967	299 (30.9)	1.63	(1.41, 1.88)		1.31	(1.11, 1.55)	
At operation	1,241	409 (33.0)	1.79	(1.58, 2.03)	<0.001	1.27	(1.08, 1.48)	0.001
Critical preoperative event								
No	16,212	3,640 (22.5)	1.00					
Yes	315	154 (48.9)	3.30	(2.64, 4.13)	<0.001	1.57	(1.19, 2.06)	0.002
Catheter to surgery								
Within 24 h	337	125 (37.1)	1.95	(1.55, 2.44)		1.17	(0.87, 1.57)	
>24 h this admission	7,844	1,730 (22.1)	0.93	(0.87, 1.01)		0.80	(0.72, 0.88)	
>24 h prev admission	8,346	1,939 (23.2)	1.00		<0.001	1.00		<0.001
Triple vessel disease								
No	7,800	1,697 (21.8)	1.00			1.00		
Yes	8,727	2,097 (24.0)	1.14	(1.06, 1.22)	<0.001	1.24	(1.12, 1.37)	<0.001
Left main stem disease								
No	13,370	3,014 (22.5)	1.00					
Yes	3,157	780 (24.7)	1.13	(1.03, 1.23)	<0.001	1.00	(0.90, 1.11)	0.986
Ejection fraction								
Good (50+ %)	11,869	2,362 (19.9)	1.00			1.00		
Fair (30 to 49%)	3,811	1,121 (29.4)	1.68	(1.54, 1.82)		1.22	(1.11, 1.34)	
Poor (<30%)	847	311 (36.7)	2.34	(2.02, 2.70)	<0.001	1.34	(1.13, 1.59)	<0.001
Operative priority								
Elective	10,148	2,043 (20.1)	1.00			1.00		
Urgent	6,152	1,644 (26.7)	1.45	(1.34, 1.56)		1.33	(1.20, 1.48)	
Emergency/Salvage	227	107 (47.1)	3.54	(2.71, 4.61)	<0.001	1.93	(1.34, 2.76)	<0.001
Cardiac procedures								
CABG only	10,828	2,167 (20.0)	1.00			1.00		
Valve only	2,482	553 (22.3)	1.15	(1.03, 1.27)		1.27	(1.08, 1.48)	
CABG+valve	1,723	644 (37.4)	2.39	(2.14, 2.66)		1.81	(1.59, 2.07)	
Other/multiple	1,494	430 (28.8)	1.62	(1.43, 1.82)	<0.001	1.62	(1.39, 1.89)	<0.001

CCS, Canadian Cardiovascular Society; NYHA, New York Heart Association; MI, myocardial infarction; prev, previous; CABG, coronary artery bypass graft; Haemoglobin, g/dL; GFR, mL/min.

**Table 7:** **Associations of preoperative factors with stage-3 acute kidney injury (AKI) in the Bristol and Birmingham development sample (complete case data)**

	**Number**	**Number (%)**	**Unadjusted**			**Fully adjusted**		
	**Total**	**Stage-3 AKI**	**Odds ratio**	**95% CI**	***P*** **-value**	**Odds ratio**	**95% CI**	***P*** **-value**
**Demographic factors**								
Age, years								
<60	4,056	75 (1.8)	1.00			1.00		
60 to 74	9,074	352 (3.9)	2.14	(1.66, 2.76)		1.25	(0.94, 1.66)	
≥75	3,397	224 (6.6)	3.75	(2.87, 4.89)	<0.001	1.29	(0.94, 1.78)	0.255
Sex								
Male	12,464	497 (4.0)	1.00			1.00		
Female	4,063	154 (3.8)	0.95	(0.79, 1.14)	0.575	0.45	(0.36, 0.55)	<0.001
Body mass index								
<20.0	594	31 (5.2)	1.57	(1.07, 2.31)		0.54	(0.35, 0.84)	
0.0 to 24.9	4,209	177 (4.2)	1.25	(1.03, 1.53)		0.83	(0.67, 1.03)	
25.0 to 29.9	7,422	251 (3.4)	1.00			1.00		
30.0 to 34.9	3,258	137 (4.2)	1.25	(1.01, 1.55)		1.79	(1.42, 2.26)	
35.0+	1,044	55 (5.3)	1.59	(1.18, 2.14)	0.006	2.60	(1.85, 3.65)	<0.001
Smoking status								
Never smoked	5,321	193 (3.6)	1.00			1.00		
Ex-smoker	9,499	402 (4.2)	1.17	(0.99, 1.40)		1.11	(0.92, 1.35)	
Current smoker	1,707	56 (3.3)	0.90	(0.67, 1.22)	0.061	1.35	(0.97, 1.88)	0.191
**Pre-operative factors**								
Angina								
No angina	3,083	176 (5.7)	1.00			1.00		
CCS 1	1,859	76 (4.1)	0.70	(0.53, 0.93)		0.79	(0.59, 1.06)	
CCS 2	4,965	142 (2.9)	0.49	(0.39, 0.61)		0.68	(0.52, 0.89)	
CCS 3	3,834	135 (3.5)	0.60	(0.48, 0.76)		0.74	(0.56, 0.98)	
CCS 4	2,786	122 (4.4)	0.76	(0.60, 0.96)	<0.001	0.79	(0.59, 1.06)	0.047
Dyspnoea								
NYHA 1	3,583	87 (2.4)	1.00			1.00		
NHYA 2	7,223	217 (3.0)	1.24	(0.97, 1.60)		1.02	(0.78, 1.32)	
NYHA 3	4,823	242 (5.0)	2.12	(1.66, 2.72)		1.23	(0.94, 1.63)	
NYHA 4	898	105 (11.7)	5.32	(3.96, 7.14)	<0.001	1.47	(1.02, 2.10)	0.063
Previous MI								
0	10,457	379 (3.6)	1.00			1.00		
1	4,928	200 (4.1)	1.12	(0.94, 1.34)		1.06	(0.86, 1.31)	
≥2	1,142	72 (6.3)	1.79	(1.38, 2.32)	<0.001	1.18	(0.86, 1.61)	0.581
Previous operations								
0	15,835	596 (3.8)	1.00			1.00		
1+	692	55 (7.9)	2.21	(1.66, 2.94)	<0.001	1.59	(1.15, 2.19)	0.006
Preoperative diabetes								
No	13,419	443 (3.3)	1.00			1.00		
Yes	3,108	208 (6.7)	2.10	(1.77, 2.49)	<0.001	1.80	(1.48, 2.18)	<0.001
Peripheral vascular disease								
No	14,924	554 (3.7)	1.00			1.00		
Yes	1,603	97 (6.1)	1.67	(1.34, 2.09)	<0.001	1.07	(0.84, 1.37)	0.582
Pulmonary disease								
No	14,490	548 (3.8)	1.00			1.00		
Yes	2,037	103 (5.1)	1.35	(1.09, 1.68)	0.006	1.02	(0.81, 1.28)	0.889
Neurological disease								
No	15,093	579 (3.8)	1.00			1.00		
Yes	1,434	72 (5.0)	1.33	(1.03, 1.70)	0.028	0.95	(0.72, 1.24)	0.685
Hypertension								
No	5,707	196 (3.4)	1.00			1.00		
Yes	10,820	455 (4.2)	1.23	(1.04, 1.46)	0.016	1.00	(0.83, 1.22)	0.972
Haemoglobin								
<10.0	309	45 (14.6)	5.79	(4.16, 8.07)		2.34	(1.59, 3.42)	
10.0 to 11.9	2,080	202 (9.7)	3.66	(3.07, 4.36)		2.17	(1.78, 2.66)	
12.0+	14,138	404 (2.9)	1.00		<0.001	1.00		<0.001
Glomerular filtration rate								
<30.0	538	98 (18.2)	8.89	(6.82, 11.59)		8.64	(6.14, 12.15)	
30.0 to 59.9	6,052	337 (5.6)	2.35	(1.95, 2.83)		2.37	(1.91, 2.94)	
60.0 to 89.9	7,199	176 (2.4)	1.00			1.00		
90+	2,738	40 (1.5)	0.59	(0.42, 0.84)	<0.001	0.46	(0.32, 0.67)	<0.001
Heparin or nitrates								
None	14,319	510 (3.6)	1.00			1.00		
Within a week	967	50 (5.2)	1.48	(1.10, 1.99)		1.00	(0.71, 1.42)	
At operation	1,241	91 (7.3)	2.14	(1.70, 2.70)	<0.001	1.20	(0.90, 1.61)	0.486
Critical preoperative event								
No	16,212	591 (3.6)	1.00					
Yes	315	60 (19.0)	6.22	(4.64, 8.34)	<0.001	2.44	(1.63, 3.66)	<0.001
Catheter to surgery								
Within 24 h	337	40 (11.9)	3.02	(2.14, 4.28)		1.28	(0.78, 2.08)	
>24 h this admission	7,844	255 (3.3)	0.75	(0.64, 0.89)		0.55	(0.45, 0.68)	
>24 h previous admission	8,346	356 (4.3)	1.00		<0.001	1.00		<0.001
Triple vessel disease								
No	7,800	315 (4.0)	1.00			1.00		
Yes	8,727	336 (3.9)	0.95	(0.81, 1.11)	0.534	1.22	(0.98, 1.52)	0.075
Left main stem disease								
No	13,370	520 (3.9)	1.00			1.00		
Yes	3,157	131 (4.1)	1.07	(0.88, 1.30)	0.499	1.09	(0.87, 1.36)	0.461
Ejection fraction								
Good (50+ %)	11,869	367 (3.1)	1.00			1.00		
Fair (30 to 49%)	3,811	204 (5.4)	1.77	(1.49, 2.11)		1.07	(0.88, 1.30)	
Poor (<30%)	847	80 (9.4)	3.27	(2.54, 4.21)	<0.001	1.32	(0.98, 1.80)	0.206
Operative priority								
Elective	10,148	323 (3.2)	1.00			1.00		
Urgent	6,152	295 (4.8)	1.53	(1.30, 1.80)		1.51	(1.20, 1.88)	
Emergency/Salvage	227	33 (14.5)	5.17	(3.52, 7.61)	<0.001	1.36	(0.75, 2.46)	0.002
Cardiac procedures								
CABG only	10,828	294 (2.7)	1.00			1.00		
Valve only	2,482	102 (4.1)	1.54	(1.22, 1.93)		1.45	(1.05, 2.01)	
CABG+valve	1,723	135 (7.8)	3.05	(2.47, 3.76)		2.02	(1.57, 2.61)	
Other/multiple	1,494	120 (8.0)	3.13	(2.51, 3.90)	<0.001	2.69	(2.02, 3.57)	<0.001

CCS, Canadian Cardiovascular Society; NYHA, New York Heart Association; MI, myocardial infarction; CABG, coronary artery bypass graft; Haemoglobin, g/dL; GFR, mL/min.

### Diagnostic utility

#### Any-stage AKI score

**Figure 1:**
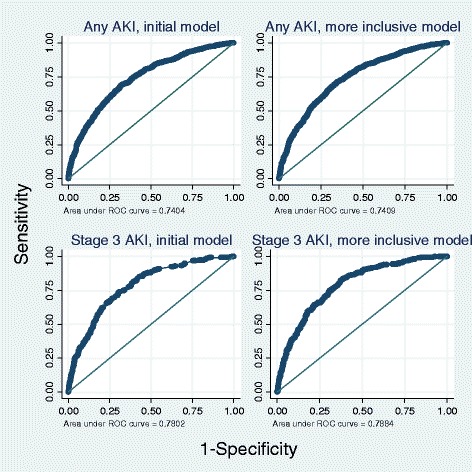
**Receiver operating characteristic curves for the any-stage acute kidney injury (AKI) and stage-3 AKI risk models in the external validation dataset (Wolverhampton data).**

**Figure 2:**
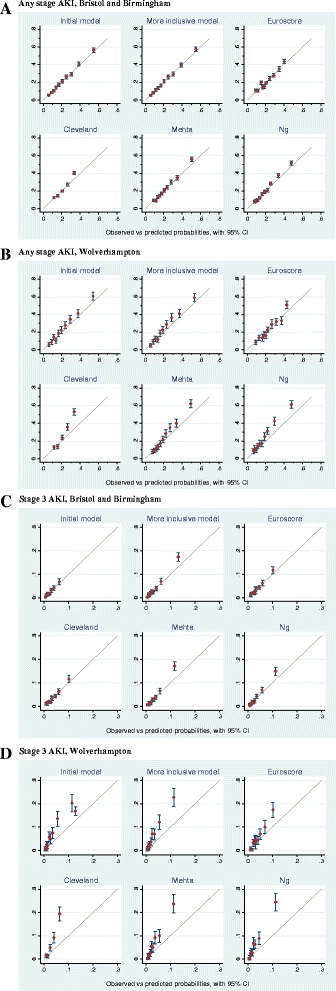
**Calibration plots of observed versus expected values for the any-stage acute kidney injury (AKI), stage-3 AKI, Cleveland, Mehta, Ng score and EuroScore in the development (Bristol, Birmingham) and validation (Wolverhampton) datasets.** **(A)** Any-stage AKI, Bristol and Birmingham. **(B)** Any-stage AKI, Wolverhampton. **(C)** Stage-3 AKI, Bristol and Birmingham. **(D)** Stage-3 AKI, Wolverhampton.

The ROC AUC for the initial model for any-stage AKI in the development sample was 0.73 (95% CI 0.72, 0.74; Figure 1 and Table 8) and was very similar in the validation sample (AUC 0.74; 95% CI 0.72, 0.76). The Hosmer Lemshow test (*P* = 0.490, Table 9) and plot of observed versus predicted any-stage AKI (Figure 2A and Table 9) demonstrated good calibration in the development sample. The initial model was less well-calibrated in the validation sample (Hosmer Lemshow *P* = 0.192, and Figure 2B). The more inclusive model did not demonstrate better discrimination when compared to the initial model (Table 8), however, it did demonstrate better calibration as evidenced by the Hosmer-Lemeshow test (*P* = 0.406 in the validation sample) and plots of observed versus predicted any-stage AKI (Figure 2B and Table 9).

**Table 8:** **Discrimination: area under ROC curves for the different scores and for different procedures (complete case data)**

	**Area under the curve (95% CI)**			
	**Development sample Bristol and Birmingham n = 16,527**		**Validation sample Wolverhampton n = 4,468**	
	**Any-stage AKI**	**Stage-3 AKI**	**Any-stage AKI**	**Stage-3 AKI**
Initial model (*P* <0.001)	0.73 (0.72, 0.74)	0.79 (0.77, 0.80)	0.74 (0.72, 0.76)	0.78 (0.75, 0.80)
More inclusive model (*P* <0.05)	0.74 (0.73, 0.75)	0.79 (0.78, 0.81)	0.74 (0.72, 0.76)	0.79 (0.76, 0.81)
**Comparison Scores**				
Euroscore	0.66 (0.65, 0.67)	0.71 (0.69, 0.73)	0.68 (0.66, 0.70)	0.73 (0.70, 0.76)
Cleveland clinic	0.65 (0.64, 0.66)	0.74 (0.72, 0.76)	0.70 (0.69, 0.72)	0.78 (0.75 0.81)
Metha score	0.71 (0.70, 0.72)	0.79 (0.77, 0.80)	0.74 (0.72, 0.76)	0.79 (0.77, 0.82)
Ng score	0.70 (0.69, 0.71)	0.77 (0.75, 0.79)	0.73 (0.71, 0.75)	0.79 (0.76, 0.82)
**Procedure type**				
Coronary artery bypass graft only	0.73 (0.72, 0.74)	0.78 (0.75, 0.81)	0.72 (0.70, 0.74)	0.78 (0.73, 0.82)
Valve only	0.72 (0.70, 0.74)	0.75 (0.70, 0.80)	0.73 (0.69, 0.77)	0.78 (0.72, 0.84)
Coronary artery bypass graft + valve	0.70 (0.66, 0.72)	0.74 (0.70, 0.79)	0.71 (0.67, 0.75)	0.70 (0.62, 0.76)
Other/multiple	0.70 (0.67, 0.73)	0.69 (0.64, 0.74)	0.70 (0.64, 0.76)	0.72 (0.62, 0.81)

*P*-values to compare diagnostic utility between our score and existing scores in validation sample for any acute kidney injury (AKI): Euroscore *P* <0.001; Cleveland clinic *P* <0.001; Metha score *P* = 0.807; Ng score *P* = 0.172. For stage-3 AKI: Euroscore *P* = 0.002; Cleveland clinic *P* = 0.801; Metha score *P* = 0.173; Ng score *P* = 0.384.

**Table 9:** **Calibration: Hosmer Lemshow tests and results of linear regression analysis of observed versus expected calibration plots**

	**Development sample Bristol and Birmingham n = 16,527**				**Validation sample Wolverhampton n = 4,468**			
	**Any-stage AKI**		**Stage-3 AKI**		**Any-stage AKI**		**Stage-3 AKI**	
**Hosmer-Lemeshow** ***P*** **-values** ^**a**^	***P***		***P***		***P***		***P***	
Initial model (*P* <0.001)	0.490		0.001		0.192		<0.001	
More inclusive model (*P* <0.05)	0.784		<0.001		0.406		<0.001	
Euroscore	<0.001		<0.001		0.333		<0.001	
Cleveland clinic	0.112		<0.001		0.141		<0.001	
Metha score	0.009		<0.001		0.136		<0.001	
Ng score	0.013		<0.001		0.174		<0.001	
	**Any-stage AKI**		**Stage-3 AKI**		**Any-stage AKI**		**Stage-3 AKI**	
**Slope and intercept** ^**b**^	**Slope**	**Intercept**	**Slope**	**Intercept**	**Slope**	**Intercept**	**Slope**	**Intercept**
Initial model (*P* <0.001)	1.045	-0.007	1.094	-0.002	1.152	-0.009	1.529	0.009
More inclusive model (*P* <0.05)	1.050	-0.008	1.334	-0.007	1.140	-0.001	2.088	-0.003
Euroscore	1.050	-0.009	1.145	-0.004	1.152	-0.038	1.789	-0.016
Cleveland clinic	1.283	-0.048	1.145	-0.004	1.927	-0.138	3.376	-0.033
Metha score	1.144	-0.029	1.513	-0.013	1.348	-0.040	2.199	-0.010
Ng score	1.130	-0.021	1.378	-0.008	1.338	-0.010	2.196	-0.004

^a^Higher values indicate better calibration. ^b^Slope and intercept from linear regression analysis of observed versus expected values analysed by decile, as plotted in Figure 2. The closer the slope is to 1, and the closer the intercept is to 0, indicates better calibration.

For the validation sample discrimination by the any-stage AKI score was better than the Euroscore (AUC 0.68; 95% CI 0.67, 0.70) and Cleveland clinic (AUC 0.70; 95% CI 0.69, 0.72), and equivalent to the Mehta Score (AUC 0.74; 0.72, 0.76) and Ng score (AUC 0.73; 95% CI 0.71, 0.75) (Table 8). Hosmer-Lemshow tests and calibration plots (Table 9 and Figure 2B) demonstrated that the more inclusive model demonstrated better calibration than each of the four comparison scores.

#### Stage-3 AKI score

In the validation sample both the initial prognostic stage-3 AKI model (0.78, 95% CI 0.75, 0.80) and the more inclusive stage-3 AKI model (0.79, 95% CI 0.76, 0.81) demonstrated good discrimination (Figure 1 and Table 8). The stage-3 AKI score had better discrimination than the Euroscore (0.73; 95% CI 0.70, 0.76) and similar discrimination to the Cleveland Clinic (0.78; 95% CI 0.75, 0.81), Metha score (0.79; 95% CI 0.77, 0.82) and Ng Score (0.79, 95% CI 0.76, 0.82) (Table 8). Plots of observed versus expected stage-3 AKI indicated that the initial stage-3 AKI model had better calibration that each of the four comparison scores, however Hosmer-Lemshow tests suggested that calibration of the stage-3 score, as well as the four comparison scores was poor (*P* <0.001) in both development and validation samples (Figure 2C, D).

#### Final model coefficients

The model coefficients for the initial model for any-stage AKI and the initial model for stage-3 AKI are shown in Table 10. Factors associated with any-stage AKI in the final model were older age, male sex, BMI >35 kg/m^2^, current smokers, higher dyspnoea categories, diabetes, peripheral vascular disease, hypertension, lower haemoglobin, lower estimated GFR, catheter to surgery within 24 hours, triple vessel disease, poor ejection fraction, emergency/salvage operations and more complex surgery. The only factor included in the stage-3 model that was not in the any-stage model was a critical preoperative event. Sensitivity analyses showed that adding two-way interactions at *P* <0.05 did not improve the diagnostic utility in the validation sample (data not shown). Further sensitivity analysis using only more recent data (after 2002) did not improve discrimination. Coefficients from imputed data for the final model were very similar to the complete case analysis (data not shown).

**Table 10:** **Model coefficients for final models (complete case data)**

	**Any-stage AKI**			**Stage-3 AKI**		
	**Odds ratio**	**95% CI**	***P*** **-value**	**Odds ratio**	**95% CI**	***P*** **-value**
Age, years						
<60	1.00					
60 to 74	1.53	(1.35, 1.73)				
≥75	2.19	(1.89, 2.54)	<0.001			
Sex						
Male	1.00			1.00		
Female	0.53	(0.48, 0.59)	<0.001	0.42	(0.34, 0.51)	<0.001
Body mass index						
<20.0	0.80	(0.64, 0.99)		0.54	(0.35, 0.82)	
20.0 to 24.9	0.80	(0.72, 0.88)		0.84	(0.68, 1.04)	
25.0 to 29.9	1.00			1.00		
30.0 to 34.9	1.75	(1.57, 1.95)		1.81	(1.44, 2.27)	
35.0+	2.08	(1.76, 2.47)	<0.001	2.71	(1.94, 3.79)	<0.001
Smoking status						
Never smoked	1.00					
Ex smoker	1.09	(1.00, 1.20)				
Current smoker	1.41	(1.22, 1.64)	<0.001			
Dyspnoea						
NYHA 1	1.00					
NHYA 2	1.05	(0.94, 1.17)				
NYHA 3	1.29	(1.14, 1.45)				
NYHA 4	1.44	(1.20, 1.74)	<0.001			
Preoperative diabetes						
No	1.00			1.00		
Yes	1.40	(1.27, 1.55)	<0.001	1.91	(1.59, 2.31)	<0.001
Peripheral vascular disease						
No	1.00					
Yes	1.35	(1.20, 1.52)	<0.001			
Hypertension						
No	1.00					
Yes	1.28	(1.17, 1.40)	<0.001			
Haemoglobin						
<10.0	1.68	(1.31, 2.17)		2.98	(2.05, 4.32)	
10.0 to 11.9	1.71	(1.53, 1.91)		2.53	(2.08, 3.07)	
12.0+	1.00		<0.001	1.00		<0.001
Glomerular filtration rate						
<30.0	4.58	(3.69, 5.69)		9.50	(6.89, 13.10)	
30.0 to 59.9	2.25	(2.04, 2.49)		2.56	(2.10, 3.13)	
60.0 to 89.9	1.00			1.00		
90.0+	0.82	(0.72, 0.95)	<0.001	0.43	(0.30, 0.62)	<0.001
Critical preoperative event						
No						
Yes				3.35	(2.35, 4.78)	<0.001
Catheter to surgery						
Within 24 h	1.22	(0.91, 1.64)		1.58	(1.03, 2.43)	
>24 hrs this admission	0.78	(0.71, 0.86)		0.71	(0.59, 0.85)	
>24 hrs previous admission	1.00		<0.001	1.00		<0.001
Triple vessel disease						
No	1.00					
Yes	1.22	(1.10, 1.34)	<0.001			
Ejection fraction						
Good (50+ %)	1.00					
Fair (30-49%)	1.24	(1.14, 1.36)				
Poor (<30%)	1.42	(1.20, 1.67)	<0.001			
Operative priority						
Elective	1.00					
Urgent	1.43	(1.30, 1.58)				
Emergency/salvage	2.45	(1.74, 3.43)	<0.001			
Cardiac procedures						
CABG only	1.00			1.00		
Valve only	1.43	(1.24, 1.65)		1.50	(1.17, 1.92)	
CABG+valve	1.89	(1.67, 2.15)		2.15	(1.72, 2.70)	
Other/multiple	1.82	(1.57, 2.11)	<0.001	2.92	(2.30, 3.72)	<0.001
Constant	0.05	(0.04, 0.06)	<0.001	0.01	(0.01, 0.02)	<0.001

Models are the initial models (variables *P* <0.001 in models adjusting for main effects). AKI, acute kidney injury; NYHA, New York Heart Association; CABG, coronary artery bypass graft; Haemoglobin, g/dL; GFR, mL/min.

## Discussion

This study has developed two new risk scores for the preoperative identification of cardiac surgery patients who are at increased risk of developing AKI. These scores were developed using a large cohort of patients from two UK cardiac centres and externally validated using data from a third UK centre. We compared the diagnostic utility of these scores to four previously published scores; Euroscore, Mehta Score, Cleveland Clinic score and the Ng score. Our risk prediction score for any-stage AKI has demonstrated better discrimination compared to the Euroscore and the Cleveland Clinic Score, and equivalent discrimination to the Mehta and Ng scores. The any-stage AKI score demonstrated better calibration than the four comparison scores. The stage-3 AKI risk prediction score demonstrated good discrimination, as did the four comparison risk scores, but these scores were less well-calibrated.

The study has important strengths. It has demonstrated the prognostic utility of the KDIGO AKI definition in a large multicentre cohort, and has confirmed the prognostic importance of milder forms of AKI, as has been identified by earlier consensus definitions [12,13]. The two risk scores we have developed are the first to our knowledge that have been designed to predict a consensus definition of AKI. The use of consensus AKI definitions as endpoints is a key element of study design that is important for standardisation of reporting and comparative analyses of trials. Furthermore, the any-stage AKI risk score is the first externally validated AKI risk prediction score that has been designed to include patients at risk of KDIGO stage-1 AKI, which we have shown to have prognostic utility in the current study. The any-stage AKI score is available as a web-based calculator [24] that is freely available to any researcher or clinician. It can be accessed by any smart phone, tablet or tabletop computer, and can be completed in less than 1 minute. Using a cutoff for the any-stage AKI score of 30% will select patients for interventional studies with a positive predictive value of 44% and a negative predictive value of 85% for AKI. We suggest that this score may be used to identify an enriched patient cohort for inclusion in clinical trials. We did not detect any advantage for our stage-3 AKI score in relation to existing scores. The stage-3 AKI score may have greater utility as a risk adjustment tool for quality assurance, or clinically to assist with informed consent. However it did not demonstrate clear advantages beyond existing score and we have not developed a web-based calculator for this score.

The study has several limitations. First, retrospective analyses of routinely collected data have limitations with respect to data quality, specifically missing data, misclassification and inconsistent data definitions between individuals and sites. To minimise these we used prospectively collected data from three clinical databases that use common, standardised definitions for clinical risk factors. The data had undergone both internal and external quality checking, had low levels of missing data, and the three sites contributing to the study are listed as among the top for data quality within the UK NACSA programme. Importantly, baseline creatinine values, defined in this study as the preoperative value obtained closest to the date of operation, were present in over 98% of patients, as would be expected in a cardiac surgery cohort where preoperative bloods are routinely taken in all but the very sickest patients. This is important; alternate definitions of baseline change the reported frequency of stage-1 AKI [25] - a key consideration in this study. The only variable for which there was a significant proportion of missing data was baseline haemoglobin, and this was restricted to a single centre. To address the limitations posed by missing data we performed our primary analysis in the complete case data. We confirmed the robustness of the models developed in the complete case analysis in imputed data. Model coefficients do not differ substantially in the imputed data, which suggests that the missing data are unlikely to have introduced bias into our model.

Second, unmeasured confounders are an important consideration in any retrospective analysis. For example, the Bristol and Birmingham databases do not routinely record patient race, and this has been found to be an important variable in some risk scores but not others [6]. This may also have affected the estimated GFR (eGFR) calculation, a key component of the score. Equations to calculate eGFR that include race, such as, for example, the modified diet in renal disease equation [26] have greater accuracy.

Third, intra and postoperative events that affect the incidence of AKI also represent confounders [10,27]. However, it was our intention to design a score that will identify patients at risk of AKI preoperatively on the basis that the most effective prevention strategies are likely to be those applied before or at the commencement of surgery rather than after injury (surgery) has occurred. Fourth, the AKI definition used did not incorporate urine output data, as defined in the KDIGO definition, as these data were not recorded. Urine output data are known to significantly alter the estimates in patients with AKI, although whether this improves the prognostic utility of the scores is unclear particularly in cardiac surgery, where perioperative urine output is closely monitored, and oliguria aggressively treated [28]. The use of creatinine-based definitions of AKI has other limitations in cardiac surgery. In the validation sample in this study 48% of patients had undergone coronary angiography within the same hospital admission and 39% were undergoing urgent inpatient surgery. Many of these patients would have sustained significant renal insults prior to surgery and this may have increased the baseline serum creatinine value.

A final limitation is the applicability of our findings to non-UK populations. We used well-defined variables that are routinely collected in UK databases, however, differences in variable definitions that occur between databases and countries may limit the wider utility of the model. This was a limitation of the two North American comparison scores. The Mehta score, in particular was not composed of variables that were routinely collected in the UK data, including MI <3 weeks, and ethnicity. The Mehta score was also developed in a selected population and both American scores excluded patients undergoing surgery without CPB, but were subsequently tested in a UK population that included a significant proportion of off-pump procedures. Conversely variable definitions were largely comparable between the UK and Australian cohorts, and the Ng score demonstrated good discrimination but poor calibration. This may reflect the non-consensus definition of AKI used in the Ng score or differences in patient populations and clinical practice between the two countries. These findings highlight the problems of risks scores developed in distinct geographic populations. The UK AKI risk scores described here may suffer from similar limitations, and we conclude that their wider utility requires independent external validation.

## Conclusion

This study has used a large multicentre cohort to develop and validate a risk prediction score for AKI stages 1 to 3. This is the only published score that predicts less severe AKI, as currently defined by the consensus KDIGO definition. We suggest that this new score core will have clinical utility for risk stratification and facilitate cohort enrichment for clinical trials of novel renoprotective interventions.

## Key messages

- AKI is a common and severe complication of cardiac surgery that contributes to morbidity, mortality and increased healthcare costs
- Previous trials of renoprotective interventions have been limited by the enrolment of low- or mixed-risk AKI cohorts, and no effective treatment has been identified thus far
- AKI risk scores are an objective and transparent way of identifying cohorts of patients at increased risk of AKI for clinical trials; however, existing scores identify only those patients who develop severe AKI requiring renal replacement therapy
- This study used data from two large cardiac surgery centres to develop a risk score that identifies all patients at risk of AKI (stages 1 to 3) with high discrimination and good calibration in an external validation dataset from a third centre
- The utility of this score is currently being prospectively validated as a cohort enrichment toll in several ongoing clinical trials

## Abbreviations

AKI: acute kidney injury
AKIN: Acute Kidney Injury Network
AUC: area under the curve
BHF: British Heart Foundation
BIPAP: bi-level positive airway pressure
BMI: body mass index
CABG: coronary artery bypass graft surgery
CCS: Canadian Cardiovascular Society
CPAP: continuous positive airway pressure
GFR: glomerular filtration rate
Hb: haemoglobin
HDU: High Dependency Unit
IABP: intra-aortic balloon pump
IQR: Interquartile range
KDIGO: Kidney Disease Improving Global Outcomes
MI: myocardial Infarction
NASCA: National Adult Cardiac Surgery Audit
NHS: National Health Service
NICOR: National Institute Clinical Outcome Research
NYHA: New York Heart Association
PATS: patient analysis and tracking system
RCT: randomised controlled trial
RIFLE: risk injury, failure, loss, end stage
ROC: receiver operating characteristic
RRT: renal replacement therapy
